# Effective educational interventions for the promotion of sexual and reproductive health and rights for school-age children in low- and middle-income countries: a systematic review protocol

**DOI:** 10.1186/s13643-020-01464-w

**Published:** 2020-09-18

**Authors:** Arone Wondwossen Fantaye, Amos Wung Buh, Dina Idriss-Wheeler, Karine Fournier, Sanni Yaya

**Affiliations:** 1 Interdisciplinary School of Health Sciences, University of Ottawa, 25 University Private, Ottawa, ON K1N 7K4 Canada; 2 Health Sciences Library, University of Ottawa, 451 Smyth Road, Ottawa, ON K1H 8M5 Canada; 3 School of International Development and Global Studies, University of Ottawa, 120 University Private, Ottawa, ON K1N 6N5 Canada; 4 The George Institute for Global Health, Oxford University, Oxford, UK

**Keywords:** Sexual, Reproductive, Health, Rights, School-aged children, Educational interventions, LMICs

## Abstract

**Background:**

Biological changes underlying the sexual and reproductive maturation of school-age children are linked with various sexual and reproductive health and rights risks. SRHR risks are predictors of poor SRHR outcomes, such as poor knowledge of sexually transmitted diseases and early sexual initiation occurring predominantly among school-age children. The aim of this proposed review, therefore, is to identify educational interventions that have proven to be effective in promoting or supporting the sexual and reproductive health and rights of school-aged children in low- and middle-income countries.

**Methods:**

A systematic review of studies on the strategies promoting the SRHR of school-aged children shall be conducted. Electronic searches will be conducted from January 2000 onwards on the following databases: MEDLINE(R) ALL (Ovid), Embase (Ovid), CINAHL (EBSCOHost), APA PsycInfo (Ovid), ERIC (Ovid), Cochrane Central Register of Controlled Trials (Ovid), Education Source (EBSCOHost), Web of Science (Clarivate Analytics), SciELO Citation Index (Clarivate Analytics), Global Health (Ovid), and Sociological Abstract (Proquest). Studies eligible for inclusion will be randomized control trials (RCTs), non-randomized trials, quasi-experimental studies (e.g., pre-post tests), and observational studies (cross-sectional and cohort studies). Peer-reviewed studies published in English and/or French and involving school-aged children 5–10 years old will be included. The primary outcomes of interest will include knowledge, awareness, or attitudes about SRHR topics. The secondary outcomes of interest will include sexual and reproductive behaviors. Two reviewers will independently screen all citations, abstract data, and full-text articles, and the methodological quality of each study will be appraised using JBI critical appraisal tools. A narrative synthesis of extracted data will be conducted.

**Discussion:**

The systematic review will synthesize the evidence on existing educational interventions targeting SRHR outcomes of school-aged children in low- and middle-income countries. It will identify which interventions have proven to be effective, and which interventions have not proven to be effective in promoting or supporting their SRHR. Review findings will provide a useful reference for policy-makers, program developers, global health leaders, and decision makers who wish to support the SRHR of school-age children.

**Systematic review registration:**

The protocol has been registered at the International Prospective Register of Systematic Reviews (PROSPERO CRD42020173158).

## Background

Middle childhood is a critical developmental period of vast physical, social, behavioral, and cognitive changes that can have a significant influence on one’s health status later in the life course [1]. It is also a period when children develop curiosity about reproduction and anatomy, experience initial physical changes (puberty) related to sexual and reproductive development, develop foundational capacities to build social relationships with the opposite sex, and experience their first sexual and romantic attractions [2, 3].

Middle-aged children learn about sexuality and reproduction, and form perceptions, attitudes, and behaviors related to what they have absorbed [4]. Their resulting perceptions, attitudes, and behaviors may contribute to healthy or unhealthy sexual and reproductive values, preferences, and decisions in subsequent life stages. Given that these decisions influence outcomes, including morbidity and poor quality of life, or death, in adolescence and adulthood, it is crucial to build a foundation for healthy and positive sexual and reproductive health outcomes in middle-aged children before they enter full sexual and reproductive maturity and activity [2].

In low- and middle-income countries (LMICs), middle childhood aligns with school-age children, which generally range from 5 to 12 years of age [1, 5, 6]. Developmental changes underlying the sexual and reproductive maturation of school-age children are linked with various sexual and reproductive health and rights risks (SRHR) [1]. SRHR risks are predictors of poor SRHR outcomes, which can include unsafe sexual practices, underage marriage, unplanned pregnancy, early childbearing, gender-based violence, sexually transmitted infections, maternity complications, and potentially death [1, 7, 8]. A review of Demographic and Health Surveys data from 55 LMICs identified insubstantial progress in delaying marriage and pregnancy, reducing unwanted pregnancies, or reducing gender inequities [9]. The United Nations Population Fund estimates that about 50 million girls in developing countries are at risk of early marriage by age 15 [10]. In 2016, early adolescent girls between the ages of 10 to 14 years had approximately 777,000 births, with 58% occurring in Africa, 28% in Asia, and 14% in Central and South America [11]. In addition, nearly a third of births by mothers under 15 years of age are unintended and unplanned [10, 11]. This is unsurprising as school-age girls entering early adolescence in LMICs are often expected to start taking on the traditional roles of women in the household, including early marriage and childbearing [12]. Child brides are still being forced into early sexual debuts and childbearing, irrespective of the adverse health risks and poor social and economic outcomes [13]. School-age boys, on the other hand, can be pressured onto a path of early and unprotected sexual activity or displays of male dominance, including sexual violence on middle-aged children or early adolescent girls [12]. Boys’ stereotypical masculinity norms and gender attitudes towards sexuality are shaped and reinforced by stereotypical masculine attributes and behaviors. These norms are often shaped by unregulated and unaccredited information, such as from peers, TV shows, and movies [12]. Gender-based violence targeting children is another rampant issue in LMICs [14]. Victims of child sexual assault tend to be school-aged children between the ages of 7 to 12 years. Given their incomplete cognitive, moral, and social development, school-aged children do not have the foundational capacity to comprehend whether they are being sexually assaulted nor can they provide informed consent [14].

Despite increasing global commitments and efforts to improve the SRHR of school-age children over the past three decades, there are still countless unmet SRHR challenges and needs for this population. Poor education and the corresponding lack of knowledge and awareness are one of the major unmet SRHR challenges and needs today [15–17]. Many school-age children in LMICs start sexual activity and reproductive maturity with only limited access to timely and adequate SRHR education and information [15, 16]. A lack of education can create barriers to accessing, receiving, and making informed decisions pertaining to SRHR, thereby increasing the likelihood of poor SRHR outcomes. These barriers can include the lack of awareness of available services, fear of privacy, and confidentiality breach to family or peers, lack of decision-making power, negative health provider attitudes, gender-based inequities, harmful sociocultural norms, as well as stigmas and taboos surrounding sexuality [10, 15, 18, 19]. Vast research evidence suggests that early educational attainment related to SRHR needs is a strong predictor of positive SRHR outcomes, including delays in sexual initiation, marriage, and pregnancy [20–22].

Developmental changes in the brain and behaviors of school-aged children, before their sexual initiation and reproductive maturation, present clear opportunities to introduce educational interventions promoting healthy and positive SHRH outcomes. There is existing systematic evidence on the effectiveness of SRHR interventions, including educational programs, targeting adolescents and young adults between ages 10 and 25 in LMICs [17, 23–36]. One manuscript in particular has reviewed the evidence on the effectiveness of sexual abuse prevention programs for school-aged children in developing countries [37]. However, it did not adhere to a specific, structured method of synthesis. There is therefore a need for a comprehensive synthesis of the evidence on the effectiveness of existing educational interventions targeting positive SRHR outcomes among school-aged children in LMICs. It is imperative to note that school-age children are a diverse group with various emerging needs. Identifying evidence-based, developmentally appropriate, and proven educational interventions on sexual and reproductive health, as well as sexual and reproductive rights, will help to address the neglected and unmet educational needs of school-age children in LMICs. While these children transition through the subsequent developmental stages, having the information and knowledge needed to make informed sexual and reproductive decisions will increase their likelihood of positive SRHR outcomes in adolescence and adulthood. Ultimately, the review findings will inform global efforts aiming to ensure access to effective SRHR information and services and aiming to reduce future risks of morbidity and mortality of school-age children.

## Objective

The objective of this study will be to conduct a systematic review of published studies that have assessed educational interventions used for promoting or supporting positive sexual and reproductive health and rights among 5 to 10 years old school-aged children in low- and middle-income countries.

### Research question

Which educational interventions have proven to be effective in promoting or supporting the sexual and reproductive health and rights of 5-10 years old school-age children in low- and middle-income countries?

## Methods

### Patient and public involvement statement

Patients were not involved in the development of this protocol.

### Protocol registration and reporting

This systematic review protocol is being reported in accordance with the reporting guidance provided by the Preferred Reporting Items for Systematic Reviews and Meta-Analyses Protocols (PRISMA-P) criteria (see Additional file 1) [38]. The review protocol has been registered within the International Prospective Register of Systematic Reviews (PROSPERO)—registration number CRD42020173158.

### Inclusion criteria

#### Population

The review will include studies that assessed educational interventions on school-aged children in LMICs. Middle childhood starts at about age five, aligning with the expected age range for school-age children. Several developmental changes, including physical and biological changes related to pubertal processes, link middle childhood to early adolescence. Health risks in middle childhood also tend to carry on into early adolescence, further connecting the two stages. Accordingly, interventional studies often aggregate middle-aged children and early adolescents into a single age range. As a result, this systematic review will likely include studies that assessed educational interventions on an age range composed of middle-aged children (5–10) and early adolescents (10–14). However, given the evidence gap, the focus will be on the 5–10 years age range. In consort, studies that solely focused on early adolescents and older (≥ 11), and that have not included children ≤ 10 years, will be excluded. The included studies must have a disaggregated age range of 5–10 years or a mean age of participants that falls between 5 and 10 years.

#### Interventions

Any educational intervention aiming to improve knowledge, awareness, attitudes, sexual behaviors, or reproductive behaviors that are relevant to the review objectives will be eligible. The review will consider studies that quantitatively evaluate the effects of educational interventions aiming to promote or support the sexual and reproductive health and rights of school-aged children in LMICs through a range of delivery channels. The educational intervention channels may include school-based interventions, conventional health services, community and outreach-based interventions, and digital media-based interventions. Interventions will be categorized by type (e.g., abstinence-only programs, comprehensive sex education programs, peer-led education), setting, and modes of delivery. Specific examples of educational interventions may include educational workshops about HIV infection, curriculum-based lessons about child sexual abuse, information about contraception access and use through social media posts, parent-led counselling about early sexual debuts and pregnancy, among others. Studies with comprehensive, composite interventions that aggregate educational interventions and non-educational interventions will be excluded if the educational intervention and its effects on an SRHR outcome are not clearly disaggregated.

#### Comparison

The review will include studies that compared the target educational intervention group(s) to no intervention groups or other intervention groups.

#### Outcomes

The review will consider studies that measure the following SRHR primary and secondary outcomes selected based on select previous work completed in the field [23–28, 37, 39]:

Primary outcomes will include knowledge, awareness, and attitudes about SRHR topics regarding HIV infection and gender-based violence (FGC, rape, assault). Examples of outcome variables include intentions to postpone sexual activity (as measured by Intentions to Postpone Sexual Activity Scale), sexual abstinence behavior skills (as measured by Sexual Abstinence Behavior Skills Scale), knowledge scores and affirmative attitude scores regarding HIV/AIDS prevention, knowledge regarding child abuse (as measured by Children’s Knowledge of Abuse Questionnaire-Revised III), self-efficacy, and curriculum specific questionnaires on personal safety or HIV/AIDS.

Secondary outcomes will include, but are not limited to, data collected in the studies that report the following: sexual initiation, number of sexual partners, prevalence or timing of adolescent pregnancy, unintended/unplanned pregnancies, child marriages, contraceptive use/safe-sex practices, gender-based violence, and sexually transmitted infections.

Effects will be considered positive if they lead to a statistically significant increase in knowledge, awareness, or attitudes, or statistically significant effects on sexual and/or reproductive behavior. The rationale for including knowledge, attitudes, and behaviors as the primary outcomes is based on the age range of the cohort (5–10 years old) for whom the primary goal of an intervention is to educate and promote positive SRHR attitudes and decisions in subsequent years when sexual activity begins [20–22].

#### Types of studies to be included

The review will include experimental and quasi-experimental studies with controlled interventions that have evaluated the effects of educational programs designed to promote or support the SRHR of school-aged children. This will include randomized control trials (RCTs), non-randomized controlled trials, quasi-randomized controlled trials, pre-post and interrupted time-series trials, and a controlled before/after comparison. Studies without a comparison group (e.g. a control intervention group) will be excluded, unless they are observational studies. Observational studies (cross-sectional and longitudinal) that examine the effectiveness of educational interventions targeting improvements in knowledge, awareness, or attitudes related to an SRHR outcome will also be included. Articles that merely indicate the prevalence of an SRHR outcome or investigate factors that influence an SRHR outcome, without implementing and testing intervention effect, will be excluded. Only studies conducted in LMICs [40] and published in peer-reviewed journals will be included in the review. In terms of language, only studies conducted in English and French will be included in the review.

### Information sources and search strategy

Electronic searches will be performed by an information specialist (KF) in the following databases: MEDLINE(R) ALL (Ovid), Embase (Ovid), CINAHL (EBSCOHost), APA PsycInfo (Ovid), ERIC (Ovid), Cochrane Central Register of Controlled Trials (Ovid), Education Source (EBSCOHost), Web of Science (Clarivate Analytics), SciELO Citation Index (Clarivate Analytics), Global Health (Ovid), and Sociological Abstract (Proquest). Studies will be identified using a combination of each of the databases’ unique subject headings and keywords (when applicable). Concepts pertaining to age (e.g., children), sexual health and rights, educational programs, and LMICs were developed for MEDLINE (see Additional file 2 for MEDLINE’s search strategy). The Cochrane LMICs filter [41] was modified to reflect the current list of LMICs identified by the World Bank [40]. The search filters for randomized controlled trials and observational studies developed by the Scottish Intercollegiate Guidelines Network (SIGN) were used and modified to include quasi-experimental designs as well [42]. All peer-reviewed publications from January 2000 onwards will be retrieved. This period accounts for a new wave and focus of studies on primary level education following the release of the Millennium Development Goals at the turn of the century.

### Screening and selection process

All the database results will be sent to Covidence (Veritas Health Innovation Ltd.), where duplicate records will be removed automatically. The articles will then be sent to the screening phase. One reviewer will independently screen all titles and abstracts, while two other reviewers will each independently screen a split of the titles and abstracts. This method will ensure that each article gets screened by two reviewers within a reasonable time frame. AWB will resolve conflicts between AWF and DIW, while DIW will resolve conflicts between AWF and AWB. The same procedure shall be repeated in screening the full-text of articles that are retained after the title and abstract screening phase. Following full-text screening, one reviewer will peruse articles relevant to the review’s objective from Prevention Science and Child Abuse and Neglect. In addition, the reference lists of included (selected) studies will be manually perused to identify additional relevant articles. Finally, articles that have cited the included (selected) full-text articles will be searched using the database Scopus to ensure the identification of additional relevant articles that may not have been identified through the database searching.

### Assessment of methodological quality

Two reviewers will independently assess the methodological quality (risk of bias) in the studies that will be selected for retrieval following full-text screening. The assessments will be done using Joanna Briggs Institute (JBI) critical appraisal tools for randomized controlled trials, quasi-experimental (non-randomized) studies, and observational studies [43] (see Additional file 3). All studies will be included in the review and weaved into the narrative description. The quality of the studies and outcome-specific evidence will be reported (as “low,” “moderate,” or “high quality”) based on the percentage of criteria met or not met. Any disagreements between the two reviewers will be settled by a third reviewer.

### Data extraction

Two reviewers will independently extract data from all included studies to minimize potential biases. Disagreements will be resolved through discussion, or a third reviewer if required. Extraction will be carried out using a standardized data extraction tool—the Joanna Briggs Institute Meta-Analysis of Statistics Assessment and Review instrument (JBI-MASARI) (see Additional file 4). The extracted data will include specific details about the study characteristics, participant characteristics, study methods, interventions (including name, type, description, timing of evaluation), outcome measures, and study results related to the review question and objectives. In the event of any missing or ambiguous data from a study, the corresponding author of the study will be contacted to retrieve missing or additional data.

### Data synthesis

We anticipate that a meta-analysis will not be appropriate given the expected heterogeneity between the studies. In particular, the studies are expected to use various designs, and to measure or report outcomes diversely, which would make it unfeasible to pool data and generate a single effect estimate. As a result, we believe a narrative summary of the effects of interventions across different studies will be the most appropriate. Following Popay’s guidance on the conduct of a narrative synthesis, the review will include a narrative synthesis of the interventional studies targeting improvements in a SRHR outcome [44]. The review will also use the synthesis without meta-analysis (SWiM) reporting guideline to help guide the reporting of the narrative synthesis [45]. The guideline has been developed to guide reviews of interventions that conduct a narrative synthesis of intervention effects.

The narrative synthesis method is ideal for synthesizing evidence from a wide range of research paradigms and study designs. The review will synthesize the evidence on the quantitatively determined effects of the various types of educational interventions using textual summaries and tabulations. Structured textual summaries will be developed for each of the individual studies, reporting the same information in a consistent manner [44]. The summaries will contextualize the extracted data and include details about the educational intervention(s), implementation strategy, and outcomes. The textual summaries will be accompanied with tables where needed. The tables will provide details of setting, study design, population characteristics, intervention, implementation strategy, outcome measures (including direction of intervention effect), and quality assessment scores. The direction of intervention effects will be categorized as one of the following: positive, statistically significant evidence of improvement on an outcome; negative, statistically significant evidence of worsening; or null, statistically non significant effect. Contrary to the more constructivist methods, the narrative synthesis does not require new, layered constructs of the evidence beyond the original data. It is thereby ideal for developing recommendations directly applicable to policy-makers, and for providing implications for future research.

### Subgroup synthesis

If a sufficient number of studies are identified, reviewers plan to examine the variability in settings (e.g. Africa vs Central/South America), study populations (e.g. boys vs girls), interventions (curriculum led vs parent led), and intervention implementation strategies (e.g., digital media vs school-based lessons). The subgroup synthesis will enable the reviewers to identify patterns within and between studies and their results. The patterns can uncover factors that may explain any differences in the effects of interventions across the individual studies [44].

### Confidence in review evidence

The review will assess the confidence and certainty of the review evidence for each outcome using the Grading of Recommendations Assessment, Development and Evaluation (GRADE) approach [46]. The overall GRADE certainty of evidence score for each outcome will be classified as “high,” “moderate,” “low,” or “very low.” The overall scores will be based on judgements of five key GRADE domains: methodological limitations, indirectness, imprecision, inconsistency, and the likelihood of publication bias [47]. Two reviewers will independently rate the certainty of evidence for the outcomes, and discrepancies will be resolved by consensus or with input from a third reviewer. The review findings will be discussed in the context of evidence certainty, strengths, and limitations of findings, along with their implications for policy initiatives, programmatic actions, and future research.

## Discussion

This systematic review will synthesize the evidence on existing educational interventions that have proven to be effective or ineffective in promoting or supporting the sexual and reproductive health and rights of school-age children. Review findings will help to form direct recommendations and inform the design or amendments to programs and policy initiatives related to children’s SRHR in LMICs. Review findings will also help to identify gaps in the existing research evidence and formulate future directions for research. Given the size of the project and the number of reviewers, we anticipate timeline-related challenges. In addition, given COVID-19 restrictions, the review team will be forced to collaborate virtually. Any amendments made to this protocol when conducting the study will be outlined in PROSPERO and in the final manuscript.

There are some potential limitations to the proposed systematic review. Firstly, since some studies will not directly use the terms “educational,” we may miss some interventions relevant to the review objectives. To mitigate this limitation, the search strategies were made to be highly comprehensive and sensitive. In addition, additional searching methods will be undertaken, such as perusing all articles that cite the included studies. Secondly, the reporting of potentially complex, multi-interventional studies that combine an educational intervention with non-educational interventions could be a limitation. To mitigate this limitation, studies that do not clearly disaggregate the educational intervention and its effects on an SRHR outcome will be excluded. Given the focus on school-age children and the potential lack of adequate educational intervention studies with comparison groups, the review may only retrieve a few eligible experimental and quasi-experimental studies. Another limitation is that observational studies assessing an intervention effect can be susceptible to major confounding biases. The review is intended for publication in a peer-reviewed journal.

## Supplementary information

**Additional file 1.** PRISMA-P 2015 Checklist.

**Additional file 2.** MEDLINE search strategy.

**Additional file 3.** JBI Critical Appraisal Checklists.

**Additional file 4.** JBI Extraction Tool.

## Data Availability

Not applicable

## Abbreviations

SRHR: Sexual and Reproductive Health and Rights
LMIC: Low- and Middle-Income Countries
PRISMA-P: Preferred Reporting Items for Systematic Reviews and Meta-Analyses Protocols
PROSPERO: International Prospective Register of Systematic Reviews
SIGN: Scottish Intercollegiate Guidelines Network
JBI: Joanna Briggs Institute
JBI-MASTARI: Joanna Briggs Institute Meta-Analysis of Statistics Assessment and Review instrument
GRADE: Grading of Recommendations Assessment, Development and Evaluation
