# Supplementary material for: Effective educational interventions for the promotion of sexual and reproductive health and rights for school-age children in low- and middle-income countries: a systematic review protocol
Source: Syst Rev. 2020 Sep 18;9:216. doi: 10.1186/s13643-020-01464-w (PMC7500715; doi:10.1186/s13643-020-01464-w)
Supplement: Supplementary file 2 — Additional file 2. MEDLINE search strategy. [file 13643_2020_1464_MOESM2_ESM.docx]

**Additional File 2**

Ovid **MEDLINE**(R) ALL 1946 to March 12, 2020 Strategy formed on March 13, 2020

1. reproductive health/ or sexual health/

2. Contraception/ or condoms/ or Family Planning Services/ or Reproductive behavior/ or Contraception behavior/

3. sexual behavior/ or safe sex/ or sexual abstinence/ or sexual harassment/ or unsafe sex/

4. pregnancy in adolescence/ or pregnancy, high-risk/ or pregnancy, unplanned/ or pregnancy, unwanted/

5. exp child abuse/ or gender-based violence/ or exp Intimate Partner Violence/

6. Rape/

7. Sexually Transmitted Diseases/ or Papillomavirus Infections/ or exp Papillomavirus Vaccines/

8. hiv infections/ or acquired immunodeficiency syndrome/ or hiv seropositivity/

9. Circumcision, Female/

10. exp Puberty/ or Menstruation/

11. Abortion, Legal/ or Abortion, Induced/

12. (abort* or miscarr* or (pregnan* adj2 terminat*)).ti,ab,kf.

13. (menstruat* or menstrual* or menarche* or prepubescen* or pubert* or pubescen*).ti,ab,kf.

14. (((sex* or reproductive*) adj2 (right* or health*)) or SRHR).ti,ab,kf.

15. (sexual* adj2 (abstinence or abstain*)).ti,ab,kf.

16. (Sex* adj2 (debut* or initiation* or initiated or early or intercourse* or behavio?r* or violence* or abus* or coercion* or maltreat* or molest* or violat*)).ti,ab,kf.

17. ((Gender* or partner* or relation* or child*) adj2 Violen*).ti,ab,kf.

18. (rape* or raping).ti,ab,kf.

19. ((Unsafe* or unprotect*) adj2 (sex* or intercourse*)).ti,ab,kf.

20. (Sexually transmitted adj2 (infection* or disease* or disorder*)).ti,ab,kf.

21. (HIV* or STI or STIs or STD or STDs or human immun?deficiency virus or human immun? deficiency virus or acquired immun?deficiency syndrome or acquired immun? deficiency syndrome or Papillomavirus or HPV).ti,ab,kf.

22. ((girl* or Female*) adj2 genital* adj2 (mutilat* or cut* or circumcis*)).ti,ab,kf.

23. ((Unplann* or unwant* or unexpect* or adolescent* or early age* or underage* or schoolchild* or prevent* or interrupt*) adj2 pregnan*).ti,ab,kf.

24. (((forced or child* or underage*) adj2 marriage*) or child* bride*).ti,ab,kf.

25. (Contracept* or condom* or family planning or (birth? adj1 (control or regulat* or spacing)) or planned parenthood or (population* adj2 (regulat* or control))).ti,ab,kf.

26. or/1-25

27. health education/ or exp consumer health information/ or exp patient education as topic/ or sex education/ or counseling/ or sex counseling/

28. education/ or curriculum/ or schools/

29. social media/ or Mobile Applications/ or Awareness/

30. computer-assisted instruction/ or interactive tutorial/ or webcast/ or Blogging/

31. program development/ or Program Evaluation/

32. ((educat* or training or counsel* or learn* or teach* or instruction* or curricul* or syllab* or awareness) adj2 (Intervention* or Program*)).ti,ab,kf.

33. ((educat* or awareness) adj2 campaign?).ti,ab,kf.

34. ((classroom* or school* or peer* or communit*) adj3 (Intervention* or Program*)).ti,ab,kf.

35. ((social media* or mhealth or mlearning or mobile health or mobile learning or facebook* or twitter* or instagram* or YouTube or "You Tube" or Google Hangout* or web* or digital* or internet* or mobile app* or app or apps or blog* or vlog* or weblog* or web-log*) adj3 (program* or intervention* or campaign?)).ti,ab,kf.

36. ((interactive or computer-assist*) adj3 (tutorial* or program* or intervention* or instruction*)).ti,ab,kf.

37. ((education* or instruction* or awareness) adj3 (tutorial* or video* or webcast* or vlog* or blog* or mobile app* or app or apps)).ti,ab,kf.

38. or/27-37

39. exp child/ or adolescent/

40. (child* or youth? or youngster* or preteen* or pre-teen* or pre-adolescen* or preadolescen* or (young adj1 (people or person*)) or p?ediatric* or juvenile* or kid or kids or girl? or boy? or schoolgirl* or schoolboy* or schoolage* or adolescen* or teen*).ti,ab,kf.

41. (school* adj2 age*).ti,ab,kf.

42. (kindergarten* or first-grader* or second-grader* or third-grader* or fourth-grader* or fifth-grader*).ti,ab,kf.

43. ((grade 1 or grade 2 or grade 3 or grade 4 or grade 5 or grade one or grade two or grade three or grade four or grade five) adj2 student?).ti,ab,kf.

44. or/39-43

45. Developing Countries/

46. exp africa/ or latin america/ or south america/ or cuba/ or dominica/ or dominican republic/ or grenada/ or haiti/ or jamaica/ or saint lucia/ or "saint vincent and the grenadines"/ or belize/ or costa rica/ or el salvador/ or guatemala/ or honduras/ or nicaragua/ or mexico/ or argentina/ or bolivia/ or brazil/ or colombia/ or ecuador/ or guyana/ or paraguay/ or peru/ or suriname/ or venezuela/ or kazakhstan/ or kyrgyzstan/ or tajikistan/ or turkmenistan/ or uzbekistan/ or exp russia/ or cambodia/ or indonesia/ or laos/ or malaysia/ or myanmar/ or philippines/ or thailand/ or timor-leste/ or vietnam/ or bangladesh/ or bhutan/ or exp india/ or afghanistan/ or iran/ or iraq/ or jordan/ or lebanon/ or syria/ or turkey/ or yemen/ or nepal/ or pakistan/ or sri lanka/ or exp china/ or "democratic people's republic of korea"/ or mongolia/ or albania/ or "bosnia and herzegovina"/ or bulgaria/ or moldova/ or montenegro/ or "republic of belarus"/ or romania/ or serbia/ or ukraine/ or comoros/ or madagascar/ or mauritius/ or papua new guinea/ or vanuatu/ or samoa/ or american samoa/ or tonga/

47. ((developing or less* developed or least developed or under developed or underdeveloped or middle income or low* income or underserved or under served or deprived or poor*) adj (countr* or nation? or population? or world or economy or economies)).ti,ab,kf.

48. (LMIC or LMICs or third world or global south).ti,ab,kf.

49. (low* adj (gdp or gnp or gross domestic or gross national)).ti,ab,kf.

50. transitional countr*.ti,ab,kf.

51. (africa* or South America* or Latin America* or Afghani* or Albania* or Algeria* or Angola* or Argentin* or Armenia* or Azerbaijan* or Bangladesh* or Benin* or Belarus* or Belize* or Bhutan* or Bolivia* or Bosnia* or Her?egovina* or Botswana* or Brazil* or Bulgaria* or Burkina Faso or Upper Volta or Burundi* or Cambodia* or Khmer* or Kampuchea* or Cameroon* or Cabo Verde* or Cape Verde* or Central African Republic or Central African* or Chad* or China or Chinese* or Colombia* or Comoro* or Congo* or Zaire or Costa Rica* or Cote d?Ivoire or Ivorian* or Ivory Coast* or Cuba* or Djibouti* or Dominica* or Dominican Republic or Dominicanos or East Timor* or Timor Leste* or Ecuador* or Egypt* or El Salvador* or Salvadorian* or Eritrea* or Ethiopia* or Fiji* or Gabon* or Gambia* or Georgia* or Ghana* or Grenad* or Guatemala* or Guinea*).ti,ab,kf.

52. (Guam* or Guiana* or Guyan* or Haiti* or Hondura* or India* or Maldiv* or Indonesia* or Iran* or Iraq* or Jamaica* or Jordan* or Kazakhs* or Kenya* or Kiribati* or Korea* or Kosov* or Kyrgyz* or Kirgizstan* or Lao PDR or Laos or Laotian* or Leban* or Lesotho* or Basotho* or Liberia* or Libya* or Madagasca* or Malagasy or Malaysia* or Sabah or Sarawak or Malawi* or Nyasaland* or Mali* or Marshall Islands or Marshallese* or Mauritania* or Mauriti* or Mexic* or Micronesia* or Moldov?a* or Mongolia* or Montenegr* or Morocc* or Mozambi* or Myanmar* or Burmese* or Burma* or Namibia* or Nepal* or Nicaragua* or Niger* or Nauru* or Papua New Guinea* or Papuan* or Pakistan* or Paraguay* or Peru* or Philippin* or Filipino* or Romania* or Russia* or Rwanda* or Saint Lucia* or St Lucia* or Saint Vincent* or Vincentian* or St Vincent* or Grenadine* or Samoa* or Sao Tome* or Senegal* or Serbia* or Sierra Leone* or Sri Lanka* or Solomon Island* or Somalia* or South Africa* or Sudan* or Surinam* or Swaziland* or Swazi* or Eswatini* or LiSwati* or Syria* or Tajik* or Tanzania* or Thai* or Togo* or Tonga* or Tunisia* or Turk* or Tuvalu* or Uganda* or Ukrain* or Uzbek* or Vanuatu* or Ni-Vanuatu* or Hebride* or Venezuela* or Vietnam* or "West Bank and Gaza" or Palestinian* or Yemen* or Zambia* or Zimbabwe*).ti,ab,kf.

53. or/45-52

54. 26 and 38 and 44 and 53

55. exp clinical studies as topic/ or Random Allocation/ or Double Blind Method/ or Single Blind Method/ or clinical trial/ or PLACEBOS/

56. (clinical study or clinical trial, phase i or clinical trial, phase ii or clinical trial, phase iii or clinical trial, phase iv or controlled clinical trial or randomized controlled trial or multicenter study or clinical trial).pt.

57. ((clinical adj trial$) or controlled trial*).tw.

58. ((singl$ or doubl$ or treb$ or tripl$) adj (blind$3 or mask$3)).tw.

59. placebo$.tw.

60. (allocated adj2 random$).tw.

61. Epidemiologic studies/ or exp case control studies/ or exp cohort studies/ or Cross-sectional studies/ or controlled before-after studies/ or interrupted time series analysis/

62. (comparative study or evaluation study or observational study).pt.

63. (Case control or (before adj1 after) or interrupted time serie* or (pre* adj2 post*)).tw.

64. (cohort adj (study or studies)).tw.

65. Cohort analy$.tw.

66. (Follow up adj (study or studies)).tw.

67. (observational adj (study or studies)).tw.

68. (Longitudinal or quasi-experimental* or quasiexperimental* or Retrospective or Nonequivalent Group* or Regression discontinuity or Instrumental variable or Natural experiment*).tw.

69. Cross sectional.tw.

70. or/55-69

71. (case reports or letter or historical article or editorial).pt.

72. (case adj (study or studies or report)).ti,ab,kf.

73. or/71-72

74. 70 not 73

75. 54 and 74

76. exp animals/ not humans.sh.

77. 75 not 76

78. limit 77 to yr="2000 -Current"
