# Supplementary material for: Effective educational interventions for the promotion of sexual and reproductive health and rights for school-age children in low- and middle-income countries: a systematic review protocol
Source: Syst Rev. 2020 Sep 18;9:216. doi: 10.1186/s13643-020-01464-w (PMC7500715; doi:10.1186/s13643-020-01464-w)
Supplement: Supplementary file 3 — Additional file 3. JBI Critical Appraisal Checklists. [file 13643_2020_1464_MOESM3_ESM.docx]

**JBI Critical Appraisal Checklists**

**JBI critical appraisal checklist for randomized control trials**

Reviewer Date

Author Year Record Number

|  | Yes | No | Unclear |
| --- | --- | --- | --- |
| 1. Was the assignment to treatment groups truly random? |  |  |  |
| 2. Were participants blinded to treatment allocation? |  |  |  |
| 3. Was allocation to treatment groups concealed from the allocator? |  |  |  |
| 4.   Were the outcomes of people who withdrew described and included in the analysis? |  |  |  |
| 5.   Were those assessing the outcomes blind to the treatment allocation? |  |  |  |
| 6.   Were control and treatment groups comparable at entry? |  |  |  |
| 7.   Were groups treated identically other than for the named interventions? |  |  |  |
| 8.   Were outcomes measured in the same way for all groups? |  |  |  |
| 9.   Were outcomes measured in a reliable way? |  |  |  |
| 10. Was appropriate statistical analysis used? |  |  |  |

Overall appraisal: Include Exclude Seek further info

Comments (including reasons for exclusion):

**JBI critical appraisal checklist for non-randomized control trials**

Reviewer Date

Author Year Record Number

|  | Yes | No | Unclear |
| --- | --- | --- | --- |
| 1. Was study based on a random or pseudo- random sample? |  |  |  |
| 2. Were the criteria for inclusion in the sample clearly defined? |  |  |  |
| 3. Were confounding factors identified and strategies to deal with them stated? |  |  |  |
| 4. Were outcomes assessed using objective criteria? |  |  |  |
| 5. If comparisons are being made, was there sufficient descriptions of the groups? |  |  |  |
| 6.  Was follow up carried out over a sufficient time period? |  |  |  |
| 7. Were the outcomes of people who withdrew described and included in the analysis? |  |  |  |
| 8.  Were outcomes measured in a reliable way? |  |  |  |
| 9.  Was appropriate statistical analysis used? |  |  |  |

Overall appraisal: Include Exclude Seek further info

Comments (including reasons for exclusion):
