# Supplementary material for: Effective educational interventions for the promotion of sexual and reproductive health and rights for school-age children in low- and middle-income countries: a systematic review protocol
Source: Syst Rev. 2020 Sep 18;9:216. doi: 10.1186/s13643-020-01464-w (PMC7500715; doi:10.1186/s13643-020-01464-w)
Supplement: Supplementary file 4 — Additional file 4. JBI Extraction Tool. [file 13643_2020_1464_MOESM4_ESM.docx]

**JBI Data Extraction Form for Experimental/Observational Studies**

Reviewer Author Date

Journal Year

Record Number

**Study Method** RCT Quasi-RCT Longitudinal

Retrospective Observational Other

*Participants*

Setting

  Population

Sample size

Intervention 1 Intervention 2 Intervention 3

*Interventions*

**Intervention 1:**

**Intervention 2:**

**Intervention 3:**

**Clinical outcome measures**

| Outcome Description | Scale/measure |
| --- | --- |
|  |  |

**Study results**

Dichotomous data

| Outcome | Intervention ( )  number / total number | Intervention ( )  number / total number |
| --- | --- | --- |
|  |  |  |
|  |  |  |
|  |  |  |
|  |  |  |
|  |  |  |

Continuous data

| Outcome | Intervention ( ) mean & SD (number) | Intervention (  ) mean & SD (number) |
| --- | --- | --- |
|  |  |  |
|  |  |  |
|  |  |  |

*Authors’ conclusions:*

**Comments:**

**Source:** JBI Database of Systematic Reviews and Implementation Reports15(7):1835-1849, July 2017.
